# Identification and Verification of Potential Biomarkers in Renal Ischemia-Reperfusion Injury by Integrated Bioinformatic Analysis

**DOI:** 10.1155/2023/7629782

**Published:** 2023-02-02

**Authors:** Ziwen Pan, Yuanyuan Yang, Rui Cao, Yang Qiu, Shanglin Li, Yuanyuan Zhao, Sheng Chang, Song Chen, Zhishui Chen, Weijie Zhang, Daqiang Zhao

**Affiliations:** Institute of Organ Transplantation, Tongji Hospital, Key Laboratory of Organ Transplantation, Ministry of Education, NHC Key Laboratory of Organ Transplantation, Key Laboratory of Organ Transplantation, Chinese Academy of Medical Sciences, Tongji Medical College, Huazhong University of Science and Technology, Wuhan, China

## Abstract

**Background:**

Renal ischemia-reperfusion injury (RIRI) plays an important role in the poor prognosis of patients with renal transplants. However, the potential targets and mechanism of IRI are still unclear.

**Method:**

Differential gene expression (DEG) analysis and weighted correlation network analysis (WGCNA) were performed on the GSE27274 dataset. Pathway enrichment analysis on the DEGs was performed. To identify the hub DEGs, we constructed a protein-protein interaction (PPI) network. Finally, the hub genes were verified, and candidate drugs were screened from the DsigDB database.

**Results:**

A hundred DEGs and four hub genes (*Atf3*, *Psmb6*, *Psmb8*, and *Psmb10*) were screened out. Pathway enrichment analysis revealed that 100 DEGs were mainly enriched in apoptosis and the TNF signaling pathway. The four hub genes were verified in animal models and another dataset (GSE148420). Thereafter, a PPI network was used to identify the four hub genes (*Atf3*, *Psmb6*, *Psmb8*, and *Psmb10*). Finally, eight candidate drugs were identified as potential drugs.

**Conclusion:**

Three hub genes (*Psmb6*, *Psmb8*, and *Psmb10*) were associated with RIRI and could be potential novel biomarkers for RIRI.

## 1. Introduction

Several individuals suffer from end-stage kidney disease every year. According to Lv and Zhang [1], 2.618 million individuals underwent renal replacement therapy in 2010. In this population of individuals, 2.05 million individuals underwent dialysis, whereas the remaining patients underwent kidney transplantation. Kidney transplantation is the preferred treatment for patients with end-stage renal disease, providing a better prognosis than dialysis. During kidney transplantation, graft injury begins at the moment the graft is separated from the donor. After a brief warm ischemic period, the grafts are placed in a hypothermal preserving solution for a long cold ischemia time (CIT) [2]. Renal ischemia-reperfusion injury (RIRI) is a process during which tissues and organs undergo an initial interruption of blood flow and subsequent restoration of blood flow. In this process, tissues and organs are subjected to oxidative stress and inflammatory reaction. Early IRI induces later loss by chronic hypoxia, reduced kidney mass, graft vascular injury, and subsequent fibrosis [3]. IRI is an important factor affecting the early functional recovery of a transplanted kidney, usually manifested as acute tubular necrosis and delayed graft function (DGF) [4]. DGF is defined as the requirement for dialysis within 7 days of transplant, and it is distinguished from primary nonfunction (PNF) by the eventual recovery of renal function [5]. Prolonged CIT is an independent risk factor for DGF [6]. DGF is the most common complication after kidney transplantation and a risk factor affecting the survival rate of transplanted kidneys and patients. Clinically, IRI has been a challenge for several years, and it still remains one today. IRI is a multifactorial and intricate process involving the activation of cell death programs, endothelial dysfunction, transcriptional reprogramming, and activation of the innate and adaptive immune systems [7]. Therefore, exploration of the key molecular mechanisms and targets is needed to address the RIRI challenge.

With the help of bioinformatics, we gain new insights into the development of disease. Weighted gene coexpression network analysis (WGCNA) is a system biology method for describing the correlation patterns among genes across microarray samples [8]. WGCNA can identify modules of highly correlated genes, and these methods have been widely used in the fields of bioinformatics to identify candidate biomarkers. Gong et al. [9] performed WGCNA and constructed a protein-protein interaction (PPI) network to identify 10 DEGs in human anaplastic thyroid cancer. Zeng et al. [10] performed WGCNA and combined experimental verification to identify two hub genes in nonalcoholic fatty liver disease.

Some bioinformatic studies on RIRI have been reported. Guo et al. [11] found 10 hub genes and Hif-1*α* signaling pathways associated with RIRI. Additionally, Zhu et al. [12] found three genes and two miRNAs that may be potential targets for RIRI. By WGCNA, Lin et al. [13] found that Rplp1 and Lgals1 were associated with the development of acute kidney injury. Building on previous studies, our study further verified the accuracy of our results and found potential drugs to treat RIRI.

Above all, we focused on DEGs at both 6 hours and 24 hours after reperfusion and performed WGCNA to find genes linked with the development of IRI. Thereafter, we performed enrichment analysis for DEGs to find potential mechanisms. Finally, we performed a variation of hub genes and found potential drugs. This study sought to offer a novel insight into the pathogenesis of IRI and found potential biomarkers and drugs for treating IRI.

## 2. Materials and Methods

### 2.1. Data Information

Gene expression profiles of GSE27274 (GPL6101) and GSE148420 (GPL14746) were obtained from the Gene Expression Omnibus (GEO) database (https://www.ncbi.nlm.nih.gov/geo/) [14]. GSE27274 contains six sham group samples and 18 kidney IRI samples (six samples at 6, 24, and 120 hours after reperfusion). However, in our analysis, we used six samples at 6 and 24 hours after reperfusion. GSE148420 contains four normal samples and eight kidney IRI samples.

### 2.2. Screening Differentially Expressed Genes

All data were log-transformed, and expression matrices were normalized. The R package of “Limma” was used to identify DEGs between IRI samples and sham group samples. DEGs with |log2FoldChange| > 1 and false discovery rate (FDR) < 0.05 were selected.

### 2.3. Function Enrichment Analysis of DEGs

To further investigate the molecular mechanisms of potential differentially expressed IRGs, we used the David database (https://david.ncifcrf.gov) [15] to conduct Gene Ontology (GO) and Kyoto Encyclopedia of Genes and Genomes (KEGG) pathway analyses. GO terms and KEGG pathway with *P* value < 0.05 were considered significantly enriched.

### 2.4. Identification of IRI-Associated Modules by WGCNA and Matching Common Genes

In this study, we constructed a coexpression network for normalized gene expression data of the GSE27274 dataset using the “WGCNA” package. The soft threshold was set to 20, whereas scale-free *R*^2^ was 0.8. The minimum number of genes in modules was set as 30 genes. The R package of “VennDiagram” was used to identify the intersection of the differential genes obtained from the GEO database and significant module genes.

### 2.5. PPI Network Analysis for the Identification of Hub Genes and ROC Curve Analysis in Another Dataset

To further identify hub genes, we established a PPI network on the Search Tool for the Retrieval on Interacting Genes (STRING) (https://string-db.org/) [16], which was used to predict associations among proteins. Additionally, we set the minimum required interaction score at 0.9. Cytoscape plug-in, CytoHubba, was used to identify hub genes. The diagnostic accuracy of hub genes was tested in another dataset.

### 2.6. Animal Model

Eighteen male Wistar rats weighing 200–300 g were obtained from Beijing Vital River Laboratory Animal Technology Co., Ltd (Beijing, China). Wistar rats were subjected to ischemia/reperfusion, as described in our previous study [17]. Rats were allocated to three groups (*n* = 18) as follows: (1) the six hours after reperfusion group (*n* = 6), (2) the 24 hours after reperfusion group (*n* = 6), and (3) the sham group (*n* = 6). Rats in the 6 and 24 hours after reperfusion groups were anesthetized. Thereafter, their right kidneys were removed, and their left renal pedicles were isolated from connective tissues and clamped for 48 minutes using a vascular clamp. For those in the sham group, they underwent the same procedure but without vessel clipping. The rats were sacrificed after 24 hours, and blood and kidneys were collected for further analysis.

### 2.7. Creatinine Analysis and Hematoxylin and Eosin (H&E) Staining

An automated biochemical analyzer BS-200 (Mindray, Shenzhen, China) was used to measure the creatinine levels of the collected blood samples. The kidney specimens were embedded in paraffin, sectioned, and stained with H&E. Tubular damage was scored, as described previously [18].

### 2.8. Western Blot Analysis

Total proteins from frozen tissues were extracted using lysis buffer (P0013J, Biyuntian, China). The protein concentration of samples was measured (BCA Protein Assay Kit (Biyuntian, China)), and equal amounts of protein were loaded for electrophoresis and subsequent transfer to polyvinylidene fluoride membranes (MilliporeSigma, Burlington, MA, USA). Using 5% BSA for 1 hour, membranes were blocked, and the primary antibody was incubated overnight at 4°C. On the second day, a secondary antibody was incubated at room temperature for 1 hour. Thereafter, proteins were visualized by ECL. A list of the primary antibodies has been provided in Supplementary Table 1.

### 2.9. Real-Time PCR

Total RNA from frozen tissues was extracted using RNA-Easy™ Isolation Reagent Vazyme Cat (Vazyme, Nanjing, China). cDNA was synthesized using a reverse transcription kit (Vazyme). The mRNA levels of these genes were assessed using the StepOne software (ThermoFisher Scientific, China). *β*-Actin was used as a reference gene. Primer sequences are listed in Supplementary Table 2.

### 2.10. Prediction of Candidate Drugs for Key Genes

Approved drugs associated with RIRI were obtained from the DsigDB database [19], which was obtained from Enrichr (https://maayanlab.cloud/Enrichr/). Candidate drugs with adjusted *P* < 0.01 were screened.

### 2.11. Statistical Analysis

**Figure 1:**
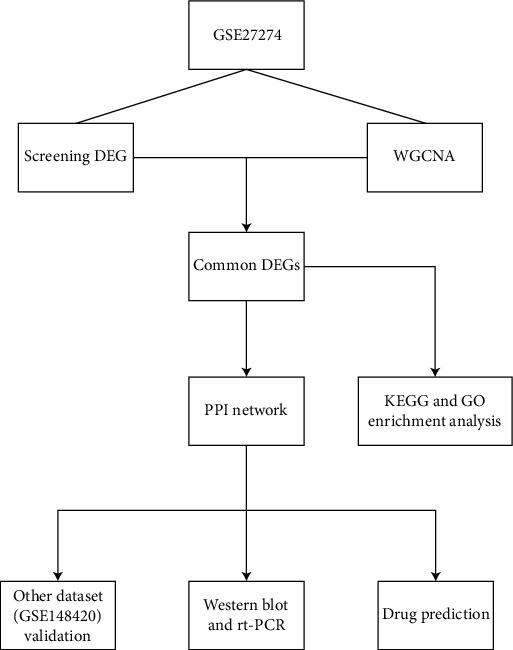
The workflow of the study.

DEGs were analyzed using the R package of “Limma” and were defined with |log2FoldChange| > 1 and false discovery rate (FDR) < 0.05. Differences between the two groups were identified using the *t*-test. *P* < 0.05 was considered statistically significant. Statistical analysis was performed using GraphPad Prism 9 software (GraphPad Software, San Diego, CA, USA).  Figure 1 depicts the workflow of the study.

## 3. Results

### 3.1. Identification of Renal IRI-Related DEGs and Associated Modules by WGCNA

**Figure 2:**
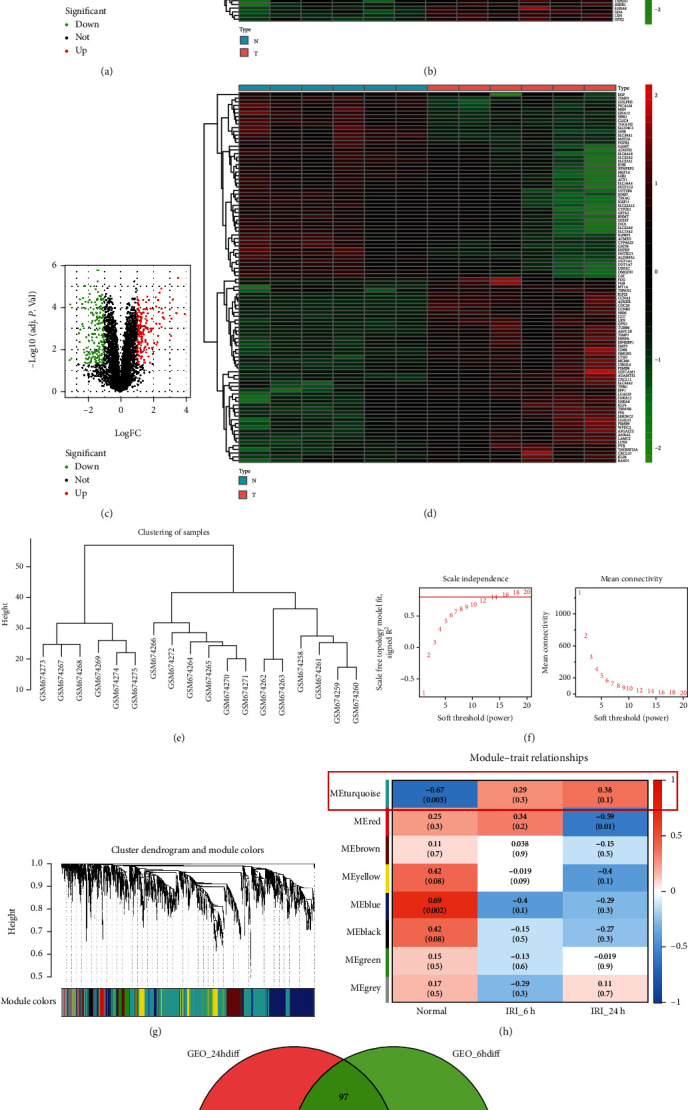
Analysis of gene expression difference and WGCNA of samples: (a, b) and (c, d) are volcano plots and heat maps for the analysis of differences between 6-hour and 24-hour reperfusion samples and sham samples, respectively; (e) sample cluster map; (f) soft thresholds for scale independence and mean connectivity; (g) the cluster dendrogram of genes; (h) module-trait relationship plotters. Each cell has a correlation coefficient and *P* value. (i) Venn plot of DEGs and turquoise module genes between 6-hour and 24-hour reperfusion samples and normal samples.

Differences in gene expression between 6-hour and 24-hour reperfusion samples and normal samples were analyzed (Figures 2(a)–2(d)). The WGCNA package was used to construct coexpression networks (Figures 2(e)–2(h)) for the GSE27274 dataset. A soft threshold *β* of 14 (Figure 2(f)) was highly suitable for constructed gene modules. Among all modules, the turquoise modules (Figure 2(h)) with 1311 genes were the most relevant for the development of renal IRI. To further identify the DEGs associated with the development of renal IRI, we drew Venn diagrams to identify intersecting genes (Figure 2(i), Table 1).

### 3.2. Functional Enrichment Analysis of DEGs

**Figure 3:**
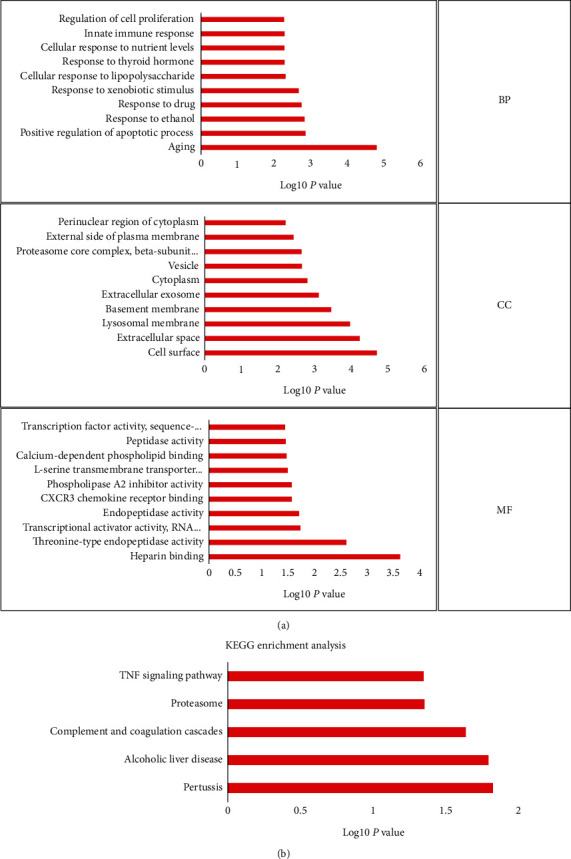
Functional enrichment analysis of DEGs: (a) GO enrichment analysis of DEGs; (b) KEGG enrichment analysis of DEGs. BP: biological process; CC: cellular component; MF: molecular function.

GO (Figure 3(a), Table 2) and KEGG (Figure 3(b), Table 3) enrichment analyses of these DEGs showed aging, cell surface, and heparin binding as the most common biological terms for biological process, cellular component, and molecular function. Moreover, pertussis was the most enriched term in the KEGG pathway analysis.

### 3.3. Constructed PPI Network for Hub Gene Identification

**Figure 4:**
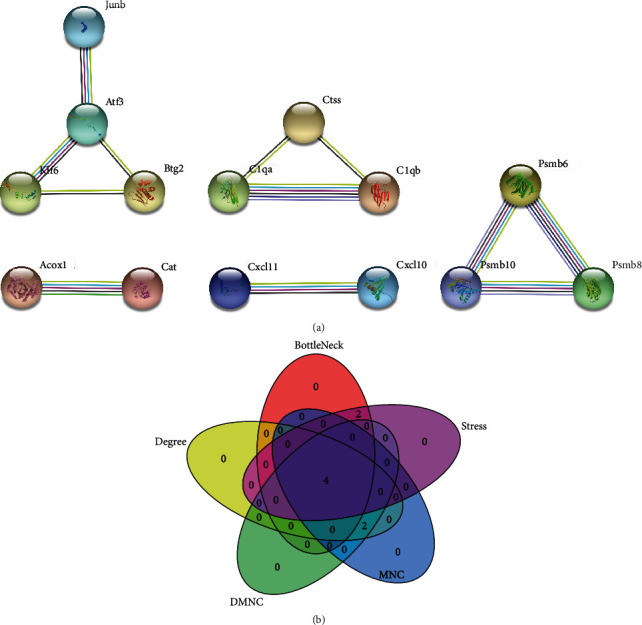
Building a PPI network of the DEGs and screening out hub genes: (a) PPI network exported from the STRING database; (b) Venn diagram for identification of hub genes by combining 5 algorithms of Cytohubba plug-in.

To construct a PPI network, we uploaded 100 DEGs to the STRING database (version: 11.5; https://cn.string-db.org/) (Figure 4(a)). Thereafter, we analyzed the top six hub genes using the Cytoscape plug-in. Based on five algorithms of the Cytohubba plug-in (Figure 4(b)), four genes (*Psmb6*, *Psmb8*, *Psmb10*, and *Atf3*) were identified as key genes of RIRI. These genes were selected for further validation in the RIRI model.

### 3.4. Constructed Rat RIRI Model

**Figure 5:**
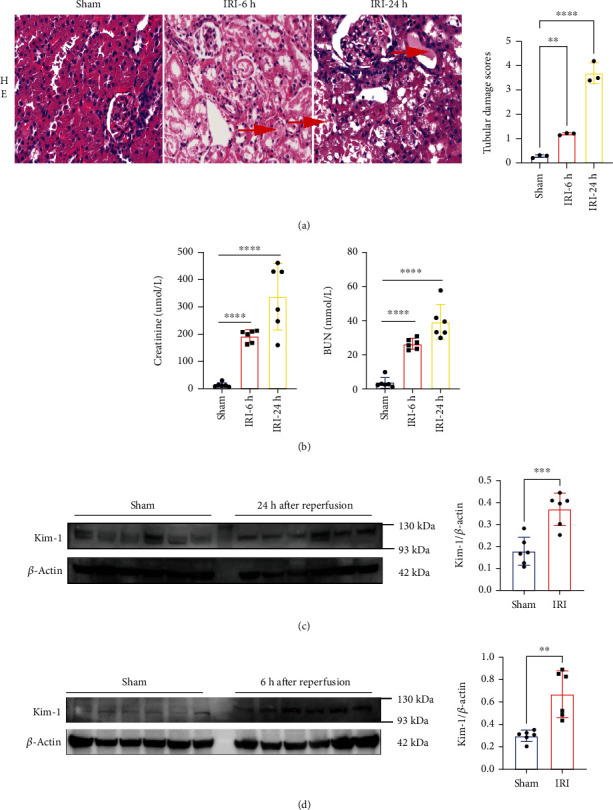
Results of animal model: (a) hematoxylin and eosin (H&E) staining (200x, left) and tubular injury scores (right). The arrow indicates that the injury was serious. (b) The levels of serum creatinine and blood urea nitrogen (BUN) in different groups. (c, d) Western blot analysis of Kim-1 expression in different groups (^∗∗∗∗^*P* < 0.0001;  ^∗∗∗^*P* < 0.001;  ^∗∗^*P* < 0.01).

As shown in Figure 5(a), H&E staining of rat kidneys helped in finding the RIRI group (6 and 24 hours after reperfusion groups), which had significant proximal tubular injury. Concurrently, serum creatinine and BUN levels were significantly higher in the RIRI group than in the sham group (Figure 5(b)). Additionally, the expression of kidney injury molecule-1 (Kim-1) was positively correlated with the severity of RIRI. Kim-1 was more highly expressed in the RIRI group than in the sham group (Figures 5(c) and 5(d)). Altogether, these results indicated that the RIRI model was successfully established.

### 3.5. External Validation and Verification of Hub Genes

**Figure 6:**
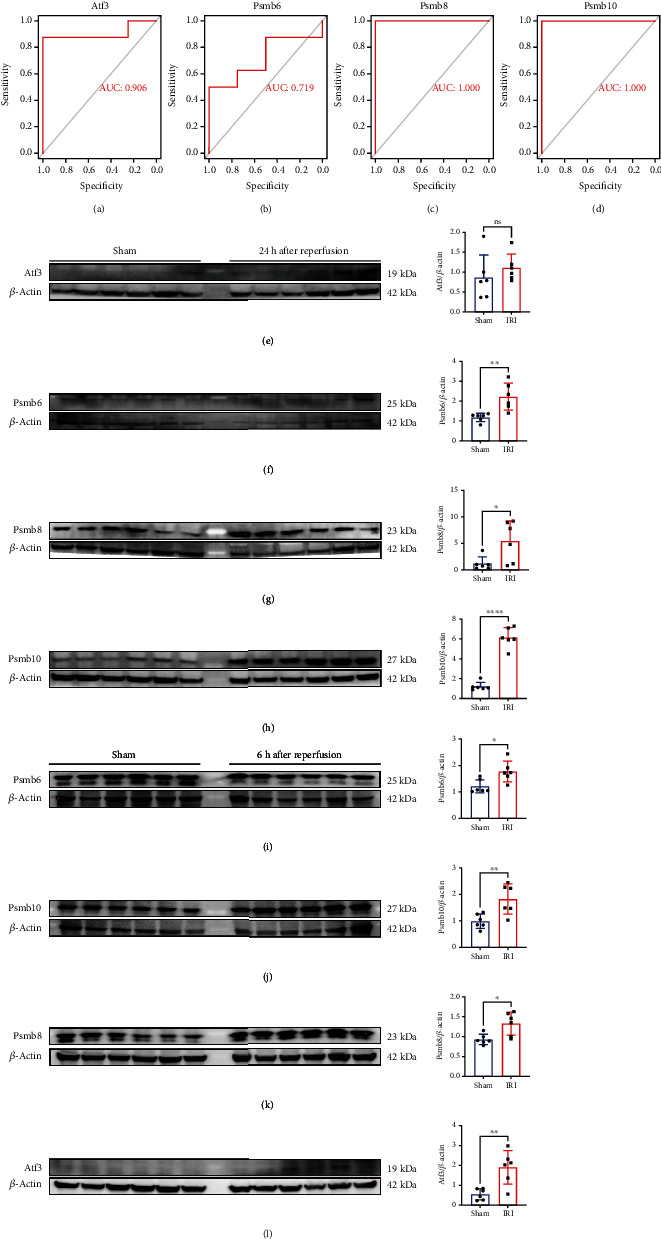
ROC curve to verify gene expression in the GSE148420 dataset and western blot in the animal model: (a–d) ROC curve of four genes in the GSE148420 dataset; (e–h) the expression of four proteins in different groups (^∗^*P* < 0.05;  ^∗∗^*P* < 0.01;  ^∗∗∗∗^*P* < 0.0001).

We performed ROC curve analysis to predict the values of four hub genes. The AUC value for *Psmb6*, *Psmb8*, *Psmb10*, and *Atf3* was 0.719, 1, 1, and 0.906, respectively, in the GSE148420 dataset (Figures 6(a)–6(d)). Consistent with our analysis of GSE27274, these four hub genes were highly expressed in the RIRI group (6 and 24 hours after reperfusion groups). However, due to lack of statistical significance, they were placed in Figures S1(a)–1(h). Additionally, we performed western blot analysis and found that *Psmb6*, *Psmb8*, and *Psmb10* were highly expressed in the RIRI group (Figures 6(f)–6(k)), whereas *Atf3* was highly expressed in the 6 hours after reperfusion group (Figures 6(e) and 6(l)).

### 3.6. Identification of Candidate Drugs Associated with Renal IRI

Eight candidate drugs (Table 4) with adjusted *P* value < 0.01 were identified in the DSigDB database. Psmb6 and Psmb8 were the most common drug targets, indicating these two genes might play a key role in the development of RIRI.

## 4. Discussion

IRI is an inevitable procedure in kidney transplantation, and the severity of kidney injury has a close correlation with graft function. Despite several studies on IRI, efforts to improve long-term kidney transplant outcomes remain challenging. Only a better understanding of RIRI pathogenesis and identification of possible targets can improve the progress of grafts.

A total of 100 DEGs were identified, and pathway enrichment analyses were performed. From the GO enrichment analysis, aging, positive regulation of apoptotic process, response to ethanol, response to drug, response to xenobiotic stimulus, and cellular response to lipopolysaccharides (LPS) were the top biological processes. Cell surface, extracellular space, and lysosomal membrane were the top cellular components. Heparin binding, threonine-type endopeptidase activity, transcriptional activator activity, and RNA polymerase II transcription regulatory region sequence-specific binding were the top molecular functions. As a special form of cell death, apoptosis, characterized by cell shrinkage, DNA fragmentation, and activation of caspases, was first proposed by Kerr et al. [20]. During RIRI, tubular epithelial cells are mostly severely damaged, and kidney injury can lead to a sharp decline in renal function. LPS is the major cell wall component of Gram-negative bacteria [21]. In the classical way, activation of TLR4 by LPS requires the interaction between LPS-binding proteins, followed by a series of reactions. LPS-TLR4 binding activates downstream signaling of the TLR4 pathway, which further leads to the activation of NF-*κ*B and inflammation.

KEGG enrichment analysis was mainly enriched in the TNF signaling pathway, proteasome, complement, and coagulation cascades. The inflammatory cytokine, TNF-*α*, has been established to play an important role in IRI. Several studies [22, 23] have indicated that TNF-*α* mRNA levels were raised significantly in the RIRI group, compared with the sham group. Furthermore, Nagata et al. [24] reported that RIRI can be ameliorated by the anti-TNF-*α* agent, infliximab. The complement system, an important component of the innate immunity system, is an important participant in the immune-inflammatory reaction, which comprises >40 blood-circulating, membrane-associated, and intracellular proteins. Many reports [25, 26] have confirmed that complement is the key mediating factor of chronic renal tubulointerstitial fibrosis after IRI. Activation of the complement system involves three activation pathways: the classical pathway, alternative pathway, and lectin pathway. During RIRI, strong inflammatory complement substances with biological activity are produced by the activation of complement; they include anaphylatoxins (C3a and C5a) and the cytolytic membrane attack complex (MAC) [27].

Additionally, we constructed a PPI network and identified four hub genes: *Atf3*, *Psmb6*, *Psmb8*, and *Psmb10.* According to our western blot analysis results, the proteins of Psmb6, Psmb8, and Psmb10 were consistently highly expressed in the 6-hour and 24-hour reperfusion groups, whereas Atf3 was only highly expressed in the 6 hours after reperfusion group. Our rt-PCR results indicated a higher tendency in the RIRI group compared to the sham group. However, this tendency was not statistically significant. The lack of statistical significance was probably due to the small sample size and reduced mRNA level changes of these genes compared to protein level changes.

Psmb6, Psmb8, and Psmb10 are all *β*-subunits of the 20S proteasome core components. The proteasome complex 26S [28] consists of the 20S catalytic core and 19S regulatory particle. The 20S core catalyzes the chamber, which contains *β*-subunits (*β*1, *β*2, and *β*5), and the 19S regulatory particle binds the polyubiquitinated substrates. Proteasome 20S has been reported to be a probable biomarker of various diseases, such as renal cell carcinoma [29], psoriasis [30], and myocardial ischemia-reperfusion injury [31].

Currently, a study [32] on hypoxia-induced pulmonary vascular in psmb6 has been reported. The expression of psmb6 increased after chronic hypoxia. During RIRI, the production of a large amount of reactive oxygen species (ROS) promoted the activation of Nrf2, which in turn plays a critical role in the protection against ROS-mediated injury. The Nrf2 agonist can alleviate tunicamycin-induced endoplasmic reticulum (ER) stress and increase the expression of Psmb6 [33]. These findings were consistent with our results. Basler et al. [34] found that psmb8 inhibitor ameliorated the pathological symptoms of dextran sulfate sodium-induced colitis by reducing inflammation. Li et al. [35] found that the deletion of psmb10 attenuates ang II-induced atrial inflammation and oxidative stress. Hypoxia, inflammation, and oxidative stress, as widely known, are closely related to IRI [24, 36]. However, these three proteasome subunits have not been reported to be associated with IRI. Our results demonstrated for the first time that the expression levels of these proteins were closely related to the development of RIRI.

Based on the DSigDB database, we found some drugs that were related to these targets by screening for drugs with adjusted *P* < 0.01. These drugs are listed in Table 4. Clopamide [37] is an oral diuretic used to treat some diseases, such as cardiac failure, nephrosis, chronic kidney failure, and cirrhosis. Azacyclonol [38], a metabolite of terfenadine, is a central nervous system inhibitor. Among these drugs, carfilzomib and borterzomib [39, 40] have been reported to be associated with IRI. Both drugs are proteasome inhibitors, and their potentially therapeutic targets are Psmb6 and Psmb8. Wu et al. [39] found that carfilzomib could reverse BNIP3L degeneration and restore mitophagy to alleviate brain IRI. However, borterzomib has different effects on the outcome of IRI-indifferent organs. Liu et al. [40] found that borterzomib alleviated myocardial IRI by activating the Nrf2/HO-1 signaling pathway. Nevertheless, borterzomib exacerbates RIRI, despite decreased secretion of infiltrating T cells and proinflammatory factors [41].

This study had some limitations. First, these findings were obtained from *in vivo* experiments but not from *in vitro* experiments and clinical samples. Second, these proteins had higher expression levels in the IRI group. However, their role in RIRI is unknown. Hence, further studies on the role of hub genes in RIRI should be conducted.

## 5. Conclusions

By bioinformatic analysis, we found some novel genes associated with the development of RIRI, and these hub genes are potential therapeutic targets for RIRI. Drugs based on target prediction may improve RIRI outcomes.

## Figures and Tables

**Table 1:** Overlap of DEGs originating from 6 hours and 24 hours of reperfusion with genes from turquoise modules.

*A3GALT2*	*CLDN5*	*GSN*	*NR1I2*	*SLC25A11*
*ACOX1*	*CLIC1*	*HNF4A*	*NT5E*	*SLC34A2*
*ADAMTS1*	*CLU*	*HPN*	*PCOLCE*	*SNRPA*
*ADHFE1*	*CRY1*	*ICAM1*	*PEX11A*	*SORD*
*ALDH9A1*	*CTSD*	*IER2*	*PGPEP1*	*STX8*
*ANXA1*	*CTSS*	*IFITM3*	*PLSCR1*	*SULT1A1*
*ANXA2*	*CXCL10*	*IRF1*	*PMM1*	*TAX1BP3*
*ANXA5*	*CXCL11*	*JUNB*	*PSMB10*	*TIMP1*
*ARPC1B*	*DMGDH*	*KIF22*	*PSMB6*	*TINAG*
*ASL*	*EIF4EBP1*	*KLF6*	*PSMB8*	*TMBIM1*
*ATF3*	*EMP3*	*LAMC2*	*PVR*	*TMEM37*
*ATP6V0E2*	*EPN1*	*LCN2*	*RASD1*	*TNFRSF12A*
*BTG2*	*FBL*	*LGALS3*	*RND1*	*TNFRSF14*
*C1QA*	*FBLIM1*	*LRPAP1*	*RPP21*	*TPBG*
*C1QB*	*FBXW5*	*MAP3K1*	*RPS9*	*TPRKB*
*CAT*	*FGB*	*METRNL*	*RUNX1*	*TUBB6*
*CD14*	*GADD45G*	*MFGE8*	*SFXN1*	*UPP1*
*CD68*	*GJA4*	*MYO7A*	*SH2D4A*	*VARS2*
*CHPT1*	*GLYCAM1*	*NFS1*	*SLC17A5*	*WFDC2*
*CHRD*	*GPRC5C*	*NQO1*	*SLC1A5*	*WSB1*

**Table 2:** GO enrichment analysis.

Category	Description	GO ID	*P* value
BP	Aging	GO:0007568	1.50*E* − 05
BP	Positive regulation of apoptotic process	GO:0043065	0.001308075
BP	Response to ethanol	GO:0045471	0.00140547
BP	Response to drug	GO:0042493	0.001686102
BP	Response to xenobiotic stimulus	GO:0009410	0.002022073
BP	Cellular response to lipopolysaccharide	GO:0071222	0.004595786
BP	Response to thyroid hormone	GO:0097066	0.004957165
BP	Cellular response to nutrient levels	GO:0031669	0.004957165
BP	Innate immune response	GO:0045087	0.004957181
BP	Regulation of cell proliferation	GO:0042127	0.005093297
CC	Cell surface	GO:0009986	1.56*E* − 05
CC	Extracellular space	GO:0005615	4.72*E* − 05
CC	Lysosomal membrane	GO:0005765	8.70*E* − 05
CC	Basement membrane	GO:0005604	2.91*E* − 04
CC	Extracellular exosome	GO:0070062	6.57*E* − 04
CC	Cytoplasm	GO:0005737	0.001345772
CC	Vesicle	GO:0031982	0.001931725
CC	Proteasome core complex, beta-subunit complex	GO:0019774	0.00195821
CC	External side of plasma membrane	GO:0009897	0.003278416
CC	Perinuclear region of cytoplasm	GO:0048471	0.005507469
MF	Heparin binding	GO:0008201	2.32*E* − 04
MF	Threonine-type endopeptidase activity	GO:0004298	0.002408191
MF	Transcriptional activator activity, RNA polymerase II transcription regulatory region sequence-specific binding	GO:0001228	0.017925491
MF	Endopeptidase activity	GO:0004175	0.019055149
MF	CXCR3 chemokine receptor binding	GO:0048248	0.026128457
MF	Phospholipase A2 inhibitor activity	GO:0019834	0.026128457
MF	L-serine transmembrane transporter activity	GO:0015194	0.031272543
MF	Calcium-dependent phospholipid binding	GO:0005544	0.033045687
MF	Peptidase activity	GO:0008233	0.034029365
MF	Transcription factor activity, sequence-specific DNA binding	GO:0003700	0.035206985

**Table 3:** KEGG enrichment analysis.

Category	Term	*P* value
KEGG_pathway	Pertussis	1.50*E* − 02
KEGG_pathway	Alcoholic liver disease	1.60*E* − 02
KEGG_pathway	Complement and coagulation cascades	2.30*E* − 02
KEGG_pathway	Proteasome	4.40*E* − 02
KEGG_pathway	TNF signaling pathway	4.50*E* − 02

**Table 4:** Prediction of candidate drugs for hub genes.

Term	Adjusted *P* value	Genes
Velcade (bortezomib) BOSS	1.63*E* − 06	*PSMB6*; *ATF3*; *PSMB8*; *PSMB10*
Carfilzomib CTD 00004787	1.63*E* − 06	*PSMB6*; *PSMB8*; *PSMB10*
Carfilzomib BOSS	1.63*E* − 06	*PSMB6*; *PSMB8*; *PSMB10*
Velcade (bortezomib) TTD 00011778	2.16*E* − 06	*PSMB6*; *PSMB8*; *PSMB10*
Carfilzomib	6.66*E* − 04	*PSMB6*; *PSMB8*
Bortezomib	0.001156865	*PSMB6*; *PSMB8*
Clopamide HL60 down	0.001156865	*PSMB6*; *PSMB8*; *PSMB10*
Azacyclonol HL60 down	0.008485756	*PSMB6*; *PSMB8*; *PSMB10*

## Data Availability

All data were acquired from public databases, including the GEO database.

## Supplementary Materials

Supplementary Materials A list of the primary and secondary antibodies and primer sequences is available in Supplementary Table 1. The rt-PCR results of four genes in the sham group and the RIRI group are illustrated in Figure S1.

## References

[1] Lv J. C., Zhang L. X. (2019). Prevalence and disease burden of chronic kidney disease. *Advances in Experimental Medicine and Biology*.

[2] Zhao H., Alam A., Soo A. P., George A. J. T., Ma D. (2018). Ischemia-reperfusion injury reduces long term renal graft survival: mechanism and beyond. *EBioMedicine*.

[3] Zhang X., Zi X. Y., Hao C. (2022). New insight in the immune mechanisms in hyperuricemia after renal transplantation: a narrative review. *European Review for Medical and Pharmacological Sciences*.

[4] Jahn N., Sack U., Stehr S. (2022). The role of innate immune cells in the prediction of early renal allograft injury following kidney transplantation. *Journal of Clinical Medicine*.

[5] Siedlecki A., Irish W., Brennan D. C. (2011). Delayed graft function in the kidney transplant. *American Journal of Transplantation*.

[6] Quiroga I., McShane P., Koo D. D. H. (2006). Major effects of delayed graft function and cold ischaemia time on renal allograft survival. *Nephrology Dialysis Transplantation*.

[7] Nieuwenhuijs-Moeke G. J., Pischke S. E., Berger S. P. (2020). Ischemia and reperfusion injury in kidney transplantation: relevant mechanisms in injury and repair. *Journal of Clinical Medicine*.

[8] Langfelder P., Horvath S. (2008). WGCNA: an R package for weighted correlation network analysis. *BMC Bioinformatics*.

[9] Gong Y., Xu F., Deng L., Peng L. (2022). Recognition of key genes in human anaplastic thyroid cancer via the weighing gene coexpression network. *BioMed Research International*.

[10] Zeng F., Shi M., Xiao H., Chi X. (2021). WGCNA-based identification of hub genes and key pathways involved in nonalcoholic fatty liver disease. *BioMed Research International*.

[11] Guo A., Wang W., Shi H., Wang J., Liu T. (2019). Identification of hub genes and pathways in a rat model of renal ischemia-reperfusion injury using bioinformatics analysis of the Gene Expression Omnibus (GEO) dataset and integration of gene expression profiles. *Medical Science Monitor*.

[12] Zhu K., Zheng T., Chen X., Wang H. (2018). Bioinformatic analyses of renal ischaemia-reperfusion injury models: identification of key genes involved in the development of kidney disease. *Kidney & Blood Pressure Research*.

[13] Lin X., Li J., Tan R., Zhong X., Yang J., Wang L. (2021). Identification of hub genes associated with the development of acute kidney injury by weighted gene co-expression network analysis. *Kidney & Blood Pressure Research*.

[14] Clough E., Barrett T. (2016). The Gene Expression Omnibus database. *Methods in Molecular Biology (Clifton, NJ)*.

[15] Huang D. W., Sherman B. T., Tan Q. (2007). DAVID Bioinformatics Resources: expanded annotation database and novel algorithms to better extract biology from large gene lists. *Nucleic Acids Research*.

[16] Szklarczyk D., Gable A. L., Nastou K. C. (2021). Correction to 'the STRING database in 2021: customizable protein-protein networks, and functional characterization of user-uploaded gene/measurement sets'. *Nucleic Acids Research*.

[17] Matsumoto T., Doi S., Nakashima A., Ike T., Sasaki K., Masaki T. (2022). Upregulation of mineralocorticoid receptor contributes to development of salt-sensitive hypertension after ischemia-reperfusion injury in rats. *International Journal of Molecular Sciences*.

[18] Li Y., Xu B., Yang J. (2021). Liraglutide protects against lethal renal ischemia-reperfusion injury by inhibiting high-mobility group box 1 nuclear-cytoplasmic translocation and release. *Pharmacological Research*.

[19] Yoo M., Shin J., Kim J. (2015). DSigDB: drug signatures database for gene set analysis. *Bioinformatics (Oxford, England)*.

[20] Kerr J. F., Wyllie A. H., Currie A. R. (1972). Apoptosis: A Basic Biological Phenomenon with Wideranging Implications in Tissue Kinetics. *British Journal of Cancer*.

[21] Ciesielska A., Matyjek M., Kwiatkowska K. (2021). TLR4 and CD14 trafficking and its influence on LPS-induced pro-inflammatory signaling. *Cellular and Molecular Life Sciences*.

[22] Zou G., Zhou Z., Xi X., Huang R., Hu H. (2021). Pioglitazone ameliorates renal ischemia-reperfusion injury via inhibition of NF-*κ*B activation and inflammation in rats. *Frontiers in Physiology*.

[23] Bao N., Dai D. (2020). Dexmedetomidine protects against ischemia and reperfusion-induced kidney injury in rats. *Mediators of Inflammation*.

[24] Nagata Y., Fujimoto M., Nakamura K. (2016). Anti-TNF-*α* agent infliximab and splenectomy are protective against renal ischemia-reperfusion injury. *Transplantation*.

[25] de Vries B., Matthijsen R. A., Wolfs T. G. A. M., van Bijnen A. A. J. H. M., Heeringa P., Buurman W. A. (2003). Inhibition of complement factor C5 protects against renal ischemia-reperfusion injury: inhibition of late apoptosis and inflammation. *Transplantation*.

[26] Møller-Kristensen M., Wang W., Ruseva M. (2005). Mannan-binding lectin recognizes structures on ischaemic reperfused mouse kidneys and is implicated in tissue injury. *Scandinavian Journal of Immunology*.

[27] Arumugam T. V., Magnus T., Woodruff T. M., Proctor L. M., Shiels I. A., Taylor S. M. (2006). Complement mediators in ischemia-reperfusion injury. *Clinica Chimica Acta*.

[28] Shi C. X., Zhu Y. X., Bruins L. A. (2020). Proteasome subunits differentially control myeloma cell viability and proteasome inhibitor sensitivity. *Mol Cancer Res*.

[29] de Martino M., Hoetzenecker K., Ankersmit H. J. (2012). Serum 20S proteasome is elevated in patients with renal cell carcinoma and associated with poor prognosis. *British Journal of Cancer*.

[30] Henry L., le Gallic L., Garcin G. (2011). Proteolytic activity and expression of the 20S proteasome are increased in psoriasis lesional skin. *The British Journal of Dermatology*.

[31] Yang C., Yu P., Yang F. (2021). PSMB4 inhibits cardiomyocyte apoptosis via activating NF-*κ*B signaling pathway during myocardial ischemia/reperfusion injury. *Journal of Molecular Histology*.

[32] Wang J., Xu L., Yun X. (2013). Proteomic analysis reveals that proteasome subunit beta 6 is involved in hypoxia-induced pulmonary vascular remodeling in rats. *PLoS One*.

[33] Lee S., Hur E. G., Ryoo I. G., Jung K. A., Kwak J., Kwak M. K. (2012). Involvement of the Nrf2-proteasome pathway in the endoplasmic reticulum stress response in pancreatic *β*-cells. *Toxicology and Applied Pharmacology*.

[34] Basler M., Dajee M., Moll C., Groettrup M., Kirk C. J. (2010). Prevention of experimental colitis by a selective inhibitor of the immunoproteasome. *Journal of Immunology (Baltimore, Md: 1950)*.

[35] Li J., Wang S., Bai J. (2018). Novel role for the immunoproteasome subunit PSMB10 in angiotensin II-induced atrial fibrillation in mice. *Hypertension*.

[36] Zhang J., Zhang J., Ni H. (2021). Downregulation of XBP1 protects kidney against ischemia-reperfusion injury via suppressing HRD1-mediated NRF2 ubiquitylation. *Cell Death Discovery*.

[37] Gupta A., Zaheer M. R., Iqbal S., Roohi, Ahmad A., Alshammari M. B. (2022). Photodegradation and in silico molecular docking study of a diuretic drug: clopamide. *ACS Omega*.

[38] Ling K. H., Leeson G. A., Burmaster S. D., Hook R. H., Reith M. K., Cheng L. K. (1995). Metabolism of terfenadine associated with CYP3A(4) activity in human hepatic microsomes. *Drug Metabolism and Disposition*.

[39] Wu X., Zheng Y., Liu M. (2021). BNIP3L/NIX degradation leads to mitophagy deficiency in ischemic brains. *Autophagy*.

[40] Liu C., Zhou J., Wang B. (2021). Bortezomib alleviates myocardial ischemia reperfusion injury via enhancing of Nrf2/HO-1 signaling pathway. *Biochemical and Biophysical Research Communications*.

[41] Huber J. M., Tagwerker A., Heininger D., Mayer G., Rosenkranz A. R., Eller K. (2009). The proteasome inhibitor bortezomib aggravates renal ischemia-reperfusion injury. *American Journal of Physiology-Renal Physiology*.

