# Supplementary material for: Identification and Verification of Potential Biomarkers in Renal Ischemia-Reperfusion Injury by Integrated Bioinformatic Analysis
Source: Biomed Res Int. 2023 Feb 2;2023:7629782. doi: 10.1155/2023/7629782 (PMC9911259; doi:10.1155/2023/7629782)
Supplement: Supplementary Materials — A list of the primary and secondary antibodies and primer sequences is available in Supplementary Table 1. The rt-PCR results of four genes in the sham group and the RIRI group are illustrated in Figure S1. [file 7629782.f1.zip › Supplementary table 1 (1).docx]

| **Supplementary Table 1:** Antibodies used in this study | | | |
| --- | --- | --- | --- |
| Antibodies | Source/Cat. No. | Host | Dilution and use |
| Kim-1 | ABclonal(A2831) | Rabbit | 1:1000 |
| β-actin | Abclonal(AC038) | Rabbit | 1:50000 |
| Psmb6 | Abcam(EPR9684) | Rabbit | 1:5000 |
| Psmb8 | CST(D1K7X) | Rabbit | 1:1000 |
| Psmb10 | Abcam(EPR14902) | Rabbit | 1:5000 |
| Atf3 | BIOSS(bs-0519R) | Rabbit | 1:1000 |
