# Supplementary material for: Identification and Verification of Potential Biomarkers in Renal Ischemia-Reperfusion Injury by Integrated Bioinformatic Analysis
Source: Biomed Res Int. 2023 Feb 2;2023:7629782. doi: 10.1155/2023/7629782 (PMC9911259; doi:10.1155/2023/7629782)
Supplement: Supplementary Materials — A list of the primary and secondary antibodies and primer sequences is available in Supplementary Table 1. The rt-PCR results of four genes in the sham group and the RIRI group are illustrated in Figure S1. [file 7629782.f1.zip › Supplementary Table 2 (1).docx]

Atf3-F CCTCTAGCCGCTCTCTGGA

Atf3-R GGCAAAGGTGCTTGTTCTGG

Psmb6-F CTCAAgAAggAgggCAggTg

Psmb6-R ggAgCCATCCCgTTCCATAg

Psmb8-F AgTCgTCATggCgTTACTgg

Psmb8-R AgCTTgCACggAgAAACTgT

Psmb10-F CggTTgTggCggATAAAAgC

Psmb10-R CTgACgCAAgATACgggTgA

β-Actin-F ACCCgCgAgTACAACCTTCT

β-Actin-R gCCgTgTTCAATggggTACT
