# Supplementary figures and images for: Identification and Verification of Potential Biomarkers in Renal Ischemia-Reperfusion Injury by Integrated Bioinformatic Analysis
Source: Biomed Res Int. 2023 Feb 2;2023:7629782. doi: 10.1155/2023/7629782 (PMC9911259; doi:10.1155/2023/7629782)

After reperfusion 6 h

a

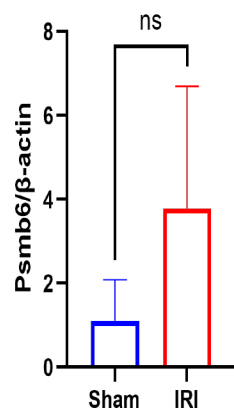

b

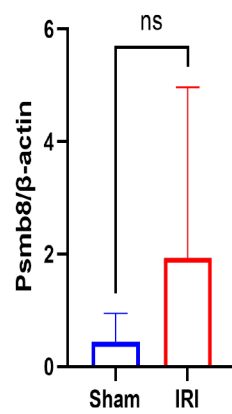

c

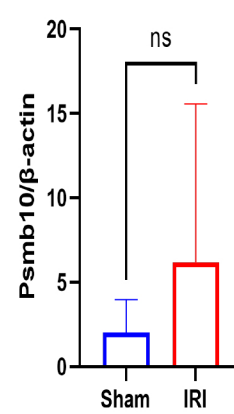

d

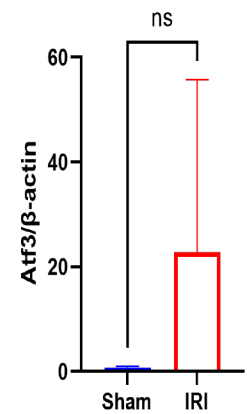

After reperfusion 24 h

e

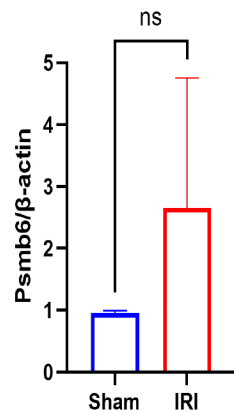

f

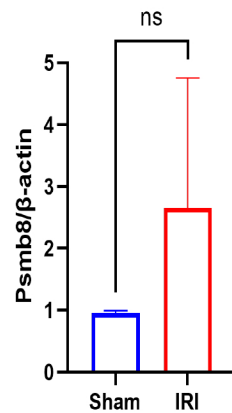

g

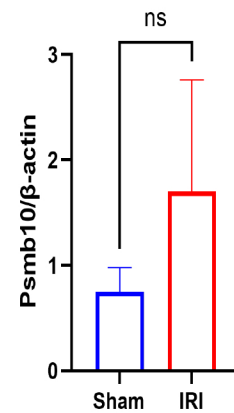

h

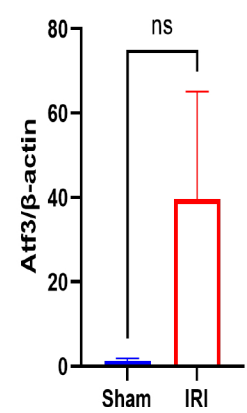

Supplement: Supplementary Materials — A list of the primary and secondary antibodies and primer sequences is available in Supplementary Table 1. The rt-PCR results of four genes in the sham group and the RIRI group are illustrated in Figure S1. [file 7629782.f1.zip › Fig S1.pdf]
